# 神经内分泌分化不是非小细胞肺癌高恶性度的指标

**DOI:** 10.3779/j.issn.1009-3419.2011.08.03

**Published:** 2011-08-20

**Authors:** 亮 戴, 宇 孙, 向红 李, 克能 陈

**Affiliations:** 100142 北京，北京大学肿瘤医院暨恶性肿瘤发病机制及转化研究教育部重点实验室胸外一科 Key Laboratory of Carcinogenesis and Translational Research (Ministry of Education), Department of Thoracic Surgery I, Peking University School of Oncology, Beijing Cancer Hospital, Beijing, 100142, China

**Keywords:** 肺肿瘤, 神经内分泌, 免疫组织化学, Lung neoplasms, Neuroendocrine, Immunohistochemistry

## Abstract

**背景与目的:**

非小细胞肺癌（Non-small cell lung cancer, NSCLC）中的神经内分泌（Neuroendocrine, NE）分化是否是恶性生物学行为与不良预后的指标，是否对化疗更敏感一直是人们关注与争论的问题。通过对NSCLC术后标本进行有关NE分化的回顾性研究，比较NE分化与NSCLC患者生存的关系，以探讨NSCLC中NE分化的临床意义。

**方法:**

按入组标准共收集274例肺癌临床资料及标本，将标本制作成组织芯片，行CgA、Syn、NCAM、Leu-7、PGP9.5和MAP-2等NE抗体染色，同时行Ki-67染色了解核增殖指数。通过不同的评分组合分析NE分化对NSCLC的预后意义。

**结果:**

不同NE评分对经手术治疗的NSCLC预后的影响均未达到统计学差异（1分、2分、≥3分*vs* 0分，*P*=0.527；0分*vs* ≥1分，*P*=0.791； < 2分*vs*≥2分，*P*=0.163； < 3分*vs*≥3分，*P*=0.293）。进一步将pTNM分期按Ⅰ期、Ⅱ期与Ⅲ期进行分层，单因素分析NE评分在各层对预后的影响，显示差异无统计学意义。围手术期化疗组不同NE评分同预后的比较无阳性结果（1分、2分、≥3分*vs*0分，*P*=0.692；0分*vs*≥1分，*P*=0.922； < 2分*vs*≥2分，*P*=0.264； < 3分*vs*≥3分，*P*=0.484）。

**结论:**

NE分化体现了部分NSCLC具有NELT结构、功能上的特点，本组研究显示NE分化同NSCLC预后无关，并非其高恶性度的指标。

## Abstract (en)

**Background and objective:**

A debate has been ongoing whether non-small cell lung cancer(NSCLC) with neuroendocrine(NE) differentiation likely indicates malignant behavior, poor prognosis, and sensitivity to chemotherapy. In response to this issue, we retrospectively investigated NE differentiation in NSCLC patients who underwent anatomical pulmonary surgery.

**Methods:**

A total of 274 patients who met the inclusion criteria through January 2000 to December 2008 were enrolled in this study because they had the detailed material and enough paraffin tumor samples for tissue microarray. The recommended antibody panel consisted of CgA, Syn, NCAM, Leu-7, PGP9.5, and MAP-2. We also counted Ki-67 in the tissues to present the nuclear proliferation index. The Kaplan–Meier estimator and the Cox proportional hazard model multivariate analysis were applied to observe the relationship between NE differentiation and postoperative survival of the patients.

**Results:**

The *Cox* analysis of different NE score combinations on the prognosis of NSCLC after surgical treatment did not reach statistical significance (score 1, score 2, and score≥3 *vs* score 0, *P*=0.527; score 0 *vs* score ≥1, *P*=0.791; score < 2 *vs* score ≥2, *P*=0.163; score < 3 *vs* score≥3, *P*=0.293). The *Kaplan–Meier* estimator did not give significant difference in the survival of NE score combinations in each pTNM layer. In the perioperative chemotherapy group, we also did not find a positive correlation for the survival analysis of NE score combinations (score 1, score 2, and score≥3 *vs* score 0, *P*=0.692; score 0 *vs* score≥1, *P*=0.922; score < 2 *vs* score ≥2, *P*=0.264; score < 3 *vs* score≥3, *P*=0.484).

**Conclusions:**

The NE differentiation of NSCLC reflects some structure and functional characteristics of NELT, although it cannot be used as an independent factor of biological behavior and survival for NSCLC patients who underwent surgery in our group.

神经内分泌分化是某些肺癌的一大特点，经典的肺神经内分泌肿瘤（neuroendocrine lung tumor, NELT）包括从典型性类癌（Typical carcinoid, TC）到小细胞肺癌（small cell lung cancer, SCLC）两个极端在内的多个具有NE形态特征的组织学类型，此不在本文研究之列。除此之外，NSCLC中存在部分肿瘤虽然不具有光镜下典型的NE细胞形态特点，但相关NE标记物染色阳性并且在电镜下可见细胞内的NE颗粒，表现出同经典NELT相似的特性。WHO将这部分具有NE分化的NSCLC归为Ⅱ类NELT^[1]^。目前，临床实践中对不具有典型光镜下NE形态结构的NSCLC不再常规进行相关NE免疫组化染色，导致人们对这一类肿瘤的诊断、治疗及预后认识不足。

本研究通过对274例手术治疗的NSCLC病例进行回顾性分析，用以探讨NE相关免疫组化结果同临床、病理特点和预后的关系。

## 1 材料和方法

### 1.1 研究对象

本研究按入组条件共收集2000年1月-2008年11月北京大学肿瘤医院单一手术组施行解剖性肺切除手术的NSCLC共274例，其中男性189例（69%），女性85例（31%），男女比例为2.2：1；年龄29岁-80岁，中位年龄61.5岁；围手术期化疗173例（63.1%）。按2009年UICC第七版肺癌TNM分期标准，Ⅰ期165例（60.2%），Ⅱ期4 8例（1 7. 5 %），Ⅲ期5 9例（2 1. 5 %），Ⅳ期2例（0.8%）（表 1）。肺叶切除术231例（84.3%），支气管/和肺血管袖状切除及成型肺切除术26例（9.5%），全肺切除术17例（6.2%）。根据2004年WHO肺癌组织学分型标准，以2位独立的资深病理医师重新阅片后，腺癌148例（54%），鳞癌103例（37.6%），大细胞未分化癌13例（4.7%），其它组织学类型10例（3.6%）。

**1:** 274例NSCLC的单因素生存分析 Univariate survival analysis of 274 NSCLCs

Item	n（%）	3-year survival±95%CI^a^	HR^c^	P^b^
Gender				0.834
Male	189（69%）	79.7%±6.5%	1.000	
Female	85（31%）	80.5%±10.4%	0.951	
Age(years)				
≥70	58（21.2%）	83.7%±10.6%	1.000	0.146
＜70	216（78.8%）	79.2%±6.3%	1.755	
≥55	202（73.7%）	81.5%±6.3%	1.000	0.484
＜55	72（26.3%）	76.6%±11.2%	1.233	
Smoking index				0.161
≥400	121（44.2%）	79.7%±7.8%	1.000	
＜400	153（55.8%）	80%±7.8%	0.724	
Location				0.534
Right lobe	143（52.2%）	80.5%±7.4%	1.000	
Left lobe	131（47.8%）	79.8%±8.2%	1.152	
Chemotherapy				0.001^**^
Yes	173（63.1%）	72.4%±8.0%	1.000	
No	101（36.9%）	89.9%±7.1%	0.352	
Surgery				0.254
Lobectomy/Sleeve resection	257（93.8%）	79.6%±5.7%	1.000	
Pneumonectomy	17（6.2%）	90.9%±17.1%	0.648	
Pathology				0.513
Adenocarcinoma	148（54%）	84.2%±7.1%	1.000	
Squamous carcinoma	103（37.6%）	76.8%±9.2%	1.433	
Large cell carcinoma	13（4.7%）	76.9%±22.9%	1.131	
Others	10（3.6%）	58.3%±38.8%	1.980	
Degree				0.037^*^
High / moderate	185（67.5%）	84.3%±5.9%	1.000	
Low/ undifferentiated	89（32.5%）	71.7%±11.0%	1.804	
pTNM				< 0.001^**^
Ⅰ	165（60.2%）	87.6%±5.7%	1.000	
Ⅱ	48（17.5%）	84.9%±11.4%	1.509	
Ⅲ	59（21.5%）	53.9%±16.3%	3.821	
Ⅳ	2（0.8%）	-	4.250	
Ki-67				0.059
≥20%	97（35.5%）	72.0%±12.2%	1.000	
＜20%	176（64.5%）	82.1%±6.3%	0.589	
^a^95%CI: 95% confidence interval; ^b^P: the *Log-rank* test result of the survival curve between 2 or more groups; ^c^HR: risk ratio, results of *Cox* regression analysis; ^*^P＜0.05, ^**^P＜0.001.				

### 1.2 免疫组化

芯片制作采用ALPHELYS的Minicore自动组织芯片仪，每组标本包含4个癌组织孔及1个癌旁组织对照孔，孔内径0.6 mm。每组至少1个癌组织孔癌细胞≥20个为有效。VENTANA自动免疫组化染色，主要试剂、来源及工作浓度详见表 2。结果由高年资病理科医师，单盲判断各点肿瘤细胞免疫组化染色情况。各标记物分别以细胞核、细胞浆或细胞膜内出现棕黄色颗粒为阳性。Ki-67半定量计算每个标本孔内阳性细胞率，取4孔平均值为阳性率。NE标记物中，一个标记物阳性记1分，阴性记0分，以每例标本6个标记物总分为0-6分计算NE评分。

**2:** 主要试剂、来源及工作浓度 Main reagents, sources and working concentrations

Item	Source	Clone	Working concentration
Rabbit anti-human chromogranin A (CgA) monoclonal antibody	ZSGB-BIO	AE1/AE3	1:400
Mouse anti-human NCAM (CD56) monoclonal antibody	ZSGB-BIO	123C3	Working solution
Mouse anti-human Leu-7 (CD57) monoclonal antibody	ZSGB-BIO	NK-1	Working solution
mouse anti-human MAP-2 monoclonal antibody	ZSGB-BIO	AP18	Working solution
Monoclonal mouse anti-human PGP9.5 antibodies	ZSGB-BIO	10A1	1:50
Rabbit anti-human synaptophysin (Syn) monoclonal antibody	ZSGB-BIO	SP11	1:100
Rabbit anti-human monoclonal antibody Ki-67	ZSGB-BIO	SP6	Working solution
3, 3 - diaminobenzidine (DAB) reagent kit	Ventana		Working solution
HRP Multimer	Ventana		Working solution

### 1.3 统计学处理

SPSS 15.0统计软件，单因素分析寻确定预后因素后，行多因素*Cox*回归分析，以NE分化为变量绘制*Kaplan-Meier*生存曲线，*Log-rank*检验生存差异。*P* < 0.05为差异有统计学意义。

## 2 结果

### 2.1 随访结果

随访结果以死亡日期和本文成稿为止（2010年4月19日），失访4例，随访率98.5%，中位随访时间为29.7个月（2.2个月-114.3个月）。

### 2.2 免疫组化结果

癌旁组织孔未见染色，各抗体染色结果见表 3。6种NE标记物，0分108例（39.4%），1分105例（38.3%），2分47例（17.2%），3分13例（4.7%），4分仅1例（0.4%），无5分和6分者。NE评分≥1分166例（60.6%），NE评分≥2分61例（22.3%），评分≥3分14例（5.1%）。Ki-67阳性率≥20%有97例（35.4%）。

**3:** 274例NSCLC的NE分化与预后单因素生存分析 NE differentiation univariate survival analysis of 274 NSCLCs

Item	n（%）	3-year survival±95%CI^a^	HR^c^	P^b^
CgA				
(+)	3（1.1%）	-	-	-
(-)	269（98.9%）	79.0%±5.7%	-	
Syn				
(+)	14（5.1%）	83.6%±21.2%	1.000	0.716
(-)	259（94.9%）	79.1%±5.7%	1.240	
NCAM				
(+)	4（1.5%）	-	-	-
(-)	270（98.5%）	79.1%±5.7%	-	
Leu-7				
(+)	71（25.9%）	90.2%±8.4%	1.000	0.063
(-)	203（74.1%）	75.7%±11.1%	1.938	
MAP-2			
(+)	108（39.4%）	80.8%±8.6%	1.000	0.768
(-)	164（60.6%）	78.3%±7.3%	1.083	
PGP9.5				
(+)	42（15.3%）	79.3%±12.5%	1.000	0.315
(-)	230（84.7%）	79.1%±6.3%	0.317	
NE score				
(1) 0	108（39.4%）	75.8%±9.4%	1.000	0.550
1	105（38.3%）	77.7%±8.8%	1.119	
2	47（17.2%）	92.4%±8.2%	0.681	
≥3	14（5.1%）	81.3%±12.4%	0.551	
(2) 0	108（39.4%）	75.8%±9.4%	1.000	0.822
≥1	166（60.6%）	82%±3.4%	0.943	
(3)＜2	213（77.7%）	76.6%±6.5%	1.000	0.170
≥2	61（22.3%）	89.3%±9.4%	0.612	
(4)＜3	260（94.9%）	79.3%±5.7%	1.000	0.407
≥3	14（5.1%）	81.3%±24.3%	0.556	
^a^95%CI：95% confidence interval；^b^P: the Log-rank test result of the survival curve between 2 or more groups；^c^HR：risk ratio，results of *Cox* regression analysis.				

### 2.3 单因素与*COX*多因素生存分析结果

将性别、年龄、吸烟指数、肿瘤部位、围手术期是否化疗、术式、病理类型、分化程度、pTNM分期及Ki-67≥20%者行单因素分析，有意义者为pTNM分期（*P* < 0.001），分化程度（*P*=0.037），而年龄（70岁，*P*=0.146），吸烟指数（*P*=0.161），Ki-67阳性率（*P*=0.059），虽未达统计学差异，但考虑其临床意义仍纳入后面*COX*分析。结果显示，CgA与NCAM阳性组样本少，且全为删失值，无法统计，其余4种均不影响NSCLC预后（表 3）。将单因素分析筛选出的各个因素分别与不同NE评分纳入*COX*回归多因素分析，建立4个数学模型，最后均得出仅TNM分期为影响本组预后唯一的独立因素。模型1结果显示NE评分0分、1分、2分与≥3分各组间生存无统计学差异（*P*=0.527）（图 1A）。模型2-模型4结果显示NE评分0分与≥1分间（*P*=0.791）（图 1B）， < 2分与≥2分间（*P*=0.163）（图 1C）， < 3分与≥3分间（*P*=0.293）（图 1D），生存均无统计学差异。

**1:**
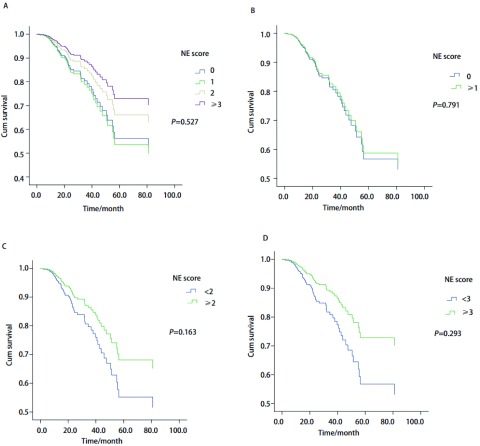
不同NE评分NSCLC的生存曲线 Cumulative *Kaplan-Meier* survival curves for different NE scores of NSCLC

### 2.4 pTNM分层生存分析结果

考虑pTNM分期为本研究中影响预后的独立因素，为最大程度排除此混杂因素干扰，进一步将pTNM分期按Ⅰ期、Ⅱ期与Ⅲ期进行分层，单因素分析NE评分在各层对预后的影响。结果显示，对于Ⅰ期组NE评分0分、1分、2分与≥3分各组间生存无统计学差异（*P*=0.735）；NE评分0分与≥1分间（*P*=0.954），＜2分与≥2分间（*P*=0.323），＜3分与≥3分间（*P*=0.558），生存亦无统计学差异。

### 2.5 化疗组生存分析结果

围手术期化疗173例，将单因素分析筛选出的性别、年龄、吸烟指数、pTNM分期、分化程度、Ki-67等因素分别与不同NE评分纳入*COX*回归多因素分析，最后结果显示无论何种NE评分组合均未对围手术期化疗患者预后产生明显影响（1分、2分、≥3分*vs* 0分：*P*=0.692；0分*vs* ≥1分：*P*=0.922； < 2分*vs* ≥2分：*P*=0. 264； < 3分*vs* ≥3分：*P*=0.484）。

## 3 讨论

NSCLC中NE分化与预后的争论由于肺部神经内分泌肿瘤中最常见的SCLC恶性度高、预后差，分类属于NSCLC中的LCNEC也同样表现出与SCLC相似的生物学特性。因此，临床惯性思维认为“NE分化”就是肺癌高度恶性生物学行为的“潜台词”。NE分化是否也是NSCLC预后不良的指标之一，学术界历来存在争论。Pelosi等^[2]^研究发现，220例Ⅰ期NSCLC患者中，NE分化阳性细胞达到5%者（至少计数2, 000个肿瘤细胞）其5年生存率要差于无NE分化和NE分化阳性细胞不足5%者（*P*=0.017）。但Sterlacci等^[3]^对405例手术的NSCLC患者研究显示，生存期与NE分化无关。Howe等^[4]^也认为不能用现有的检测NE分化的免疫化学指标CgA、Syn和NCAM对NSCLC进行预后评价。更有甚者持完全相反的观点，Schleusener等^[3]^研究发现具有NE分化患者似乎生存期更长，2项或2项以上标志物阳性的患者生存期明显长于NE阴性NSCLC患者。

本研究不支持NE分化是NSCLC高恶性度的指标本研究对6种NE标记物分别行单因素生存分析，未发现任一种抗体能对预后产生明显影响，说明不能通过单一NE标记物预测生存。同样，各种NE评分组合也未在单因素分析中显示出对预后的影响。为了排除其它混杂因素的干扰，本研究采用Cox多因素分析模型来分析NE分化与术后患者的生存关系。分析结果显示无论何种NE评分均未能对预后产生明显影响。而在多因素分析中，再次验证了pTNM分期是影响NSCLC预后的独立因素。为此，本研究分别在Ⅰ期、Ⅱ期和Ⅲ期中比较NE评分与预后的关系。发现对于Ⅰ期接受手术治疗的患者，无论何种NE评分均未显示出生存上的差异，说明对于Ⅰ期NSCLC术后患者，NE分化同预后无关。对于Ⅱ期和Ⅲ期的患者，若以至少1种抗体阳性或至少2种抗体阳性作为NE分化的标准，NE分化均同预后无关。通过不同的分层及分析方法，各组结果均未发现不同NE评分与NSCLC预后存在明显的关系，因此，我们认为，本组样本中NE分化并非影响手术治疗NSCLC预后的独立因素。

基于以上研究结果，我们认为NE分化仅仅是NE肿瘤在结构、功能上的共同特征，并非是决定此类肿瘤生物学恶性程度的因素。SCLC生物学行为高度恶性，生长迅速，早期即可出现远处转移，预后在肺癌中最差；而类癌生物学行为良好，生存时间长，同良性肿瘤类似。究其主要原因，在于SCLC属未分化癌，预后不良，而TC属高分化癌，预后也良好。当然，本组研究样本量较少，随访时间较短，尤其是单一指标阳性率太低，阳性病例绝对值太少，不除外随着样本量增大及随访时间的延长或更敏感的NE标记物的问世而出现与本结果不同的结果。但我们认为，与TNM分期、分化程度，及典型的Ⅰ类NELT肿瘤对预后的影响相比，NSCLC的NE分化至少不是一个主要的预后影响因素。

本研究不支持NE分化为NSCLC的化疗敏感性指标目前，就伴有NE分化的NSCLC是否对化疗更敏感的论点国内外研究结论也不尽一致。文献报道在影像学上NSCLC伴NE分化与化疗敏感性不相关。持此观点者为Graziano等^[5]^和Schleusener等^[6]^。但Skov等^[7]^测定了114例不能手术的肺腺癌化疗患者组织中的CgA和NSE，发现CgA阳性和阴性患者中对化疗敏感的比例分别是30%和19%。因此，认为具有NE分化的NSCLC对化疗敏感性更高。余少平等^[8]^认为NSCLC伴NE分化者多药耐药相关蛋白的阳性率较低，对化疗相对敏感。本研究中围手术期化疗共173例，结果显示无论何种NE标记物组合均未体现出显著的生存差异，但生存曲线提示NE评分越高预后越好。然而，更为确定的结论需要增大样本量及延长随访时间来验证。

从以往的研究中我们发现对于NE分化的判断并没有统一的免疫组化标准。不同的研究不仅在抗体选取、浓度配置及实验方法上存在差异，但对结果影响最大的是NE分化判定标准的不统一。鉴于此，为了尽可能减小由于以上差异所带来的选择偏倚，本研究选取了6种反映NE分化的标记物，不设定NE分化的免疫组化标准，而是通过不同的评分组合来代表不同的NE分化判定标准。通过该模型进行生存分析，解决了NE分化判定标准带来的误差，而且大大降低了以往研究抗体选择不同造成的偏倚。

## 4 结论

NSCLC的NE分化对NSCLC的远期生存没有影响，不构成为一个影响远期预后的因素。NSCLC的NE分化也许只代表着这类肿瘤细胞的形态特征。本组研究再次肯定了TNM分期与分化程度是影响预后的主要因素。同时，治疗后良好的生存率再次肯定了较早期NSCLC的治疗仍应以手术治疗为主。为更宽泛的认定NSCLC的NE分化，以便就NE分化的预后及治疗意义作出方向性研究，本研究采用数学建模，模糊NE分化判定标准，采用量化的方式（NE评分0分-6分），全面考察不同NE标记物及组合对预后的影响，方法值得借鉴。NSCLC的NE分化需要相关的免疫组织化学染色来认定。本研究所选用各单一标记物的敏感性与特异性均不能达到很好的统一。因此，如何认定这些指标的临床意义，抑或寻找新的指标仍是此项研究的重点。
